# A Qualitative Study of the Influences on Clinical Academic Physicians’ Postdoctoral Career Decision Making

**DOI:** 10.1097/ACM.0000000000002141

**Published:** 2018-01-23

**Authors:** Veronica F. Ranieri, Helen Barratt, Geraint Rees, Naomi J. Fulop

**Affiliations:** 1 **V.F. Ranieri** is research associate, Academic Careers Office, School of Life and Medical Sciences, and Department of Applied Health Research, University College London, London, United Kingdom; ORCID: http://orcid.org/0000-0003-0528-8640.; 2 **H. Barratt** is clinical senior research associate, Department of Applied Health Research, University College London, London, United Kingdom; ORCID: http://orcid.org/0000-0002-1387-137X.; 3 **G. Rees** is dean, Faculty of Life Sciences, professor of cognitive neurology, and director, Academic Careers Office, School of Life and Medical Sciences, University College London, London, United Kingdom; ORCID: http://orcid.org/0000-0002-9623-7007.; 4 **N.J. Fulop** is professor of health care organization and management, Department of Applied Health Research, University College London, London, United Kingdom; ORCID: http://orcid.org/0000-0001-5306-6140.

## Abstract

**Purpose:**

To describe the influences on clinical academic physicians’ postdoctoral career decision making.

**Method:**

Thirty-five doctoral trainee physicians from University College London took part in semistructured interviews in 2015 and 2016. Participants were asked open-ended questions about their career to date, their experiences undertaking a PhD, and their career plans post PhD. The interviews were audio-recorded and transcribed. Thematic analysis was used to generate, review, and define themes from the transcripts. Emerging differences and similarities in participants’ reasons for pursuing a PhD were then grouped to produce typologies to explore how their experiences influenced their career decision making.

**Results:**

Participants described four key reasons for undertaking a PhD, which formed the basis of the four typologies identified. These reasons included the following: to pursue a clinical academic career; to complete an extensive period of research to understand whether a clinical academic career was the desired path forward; to improve clinical career prospects; and to take a break from clinical training.

**Conclusions:**

These findings highlight the need to target efforts at retaining clinical academic physicians according to their reasons for pursuing a PhD and their subsequent experiences with the process. Those responsible for overseeing clinical training must be well informed of the long-term benefits of training academically qualified physicians. In light of current political uncertainty, universities, hospitals, and external agencies alike must increase their efforts to inspire and assuage early-career clinical academic physicians’ fears regarding their academic future.

Academic medicine is facing a shortage of clinical academic physicians.^1–3^ This shortage is attributed, in part, to academia’s inability to retain this group after they have completed their PhD training.^4^ Yet these researchers are key to the future of medicine as innovations and advances in treatment depend on a continuous supply of clinical academic physicians. The available literature suggests that a range of factors may influence their career decision making and may therefore steer us toward a solution to this shortage.

A recent scoping review we conducted of the worldwide English-language literature identified key factors that may influence clinical academic physicians’ postdoctoral career progression.^5^ Trainees who were intrinsically motivated to conduct research were more likely to pursue a clinical academic career. Those who felt excluded working in an unwelcoming environment, without access to a mentor, tended to leave academic medicine. Furthermore, those unable to secure funding or balance work and personal obligations were also more likely to leave after their PhD. Although this review was novel in that we collated the motivators and barriers experienced by aspiring clinical academic physicians and included a theoretical model to explain these experiences, the studies we included were predominantly North America focused, and we may, therefore, have overlooked important contextual influences present in the United Kingdom.

In the United Kingdom, the National Institute for Health Research developed an integrated academic training pathway, with the purpose of replenishing and retaining the clinical academic physician workforce.^6^ This pathway, now in place for over a decade, has yet to be formally evaluated. In an effort to bridge this gap, we conducted two studies. In our first study, we set out to understand the career plans of clinical academic physicians who were PhD students and their views regarding clinical lectureships, competitive postdoctoral awards that allow physicians to dedicate equal time to clinical duties and research, using an online survey.^7^ We found that half of those pursuing a PhD expressed a desire to continue in a clinical academic career and that a similar proportion also revealed little to no knowledge of clinical lectureships.

Building on these findings, in the present study, we analyzed the factors that influenced early-career clinical academic physicians’ career decision making, adopting the systems theory framework to guide our work.^8^ This framework offers a means of understanding the wide range of influences that are likely to impact career development, divided according to whether they occur within the individual, within the individual’s social system, or within the individual’s broader environment. From the influences we identified, we propose actions targeted at universities, hospitals, and external agencies like funders to improve the support provided to postdoctoral clinical academic physicians.

## Method

### Study design

We interviewed early-career clinical academic physician trainees and analyzed the data using a qualitative methodology to extract rich and meaningful narratives of the influences experienced by this group.^9^ This study was approved by the University College London (UCL) research ethics committee.

### Participants

**Figure 1:**
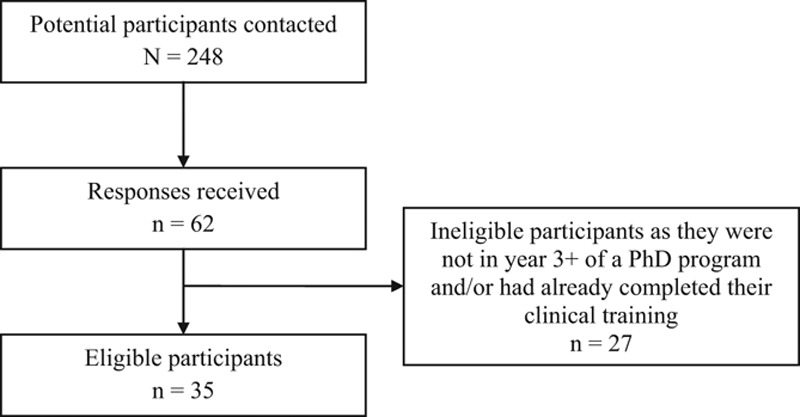
Flowchart of the participant recruitment process for a qualitative study of the influences on clinical academic physicians’ postdoctoral career decision making, University College London, 2015–2016.

A total of 35 trainees registered to practice in the United Kingdom and enrolled in a PhD program while completing their postgraduate specialty clinical training took part in an interview with a researcher from our team (V.F.R.) between November 2015 and April 2016 (see Figure 1 for recruitment details). These trainees were recruited from UCL, which is home to the United Kingdom’s largest group of clinical academic physicians and has a dedicated Academic Careers Office.^10^ Trainees were identified from a list of current and graduated doctoral students, across all clinical specialties, held by UCL’s academic registry. This list included each trainee’s date of registration, discipline of study, and, for those who had submitted their thesis, date of thesis submission. Those who had completed their clinical training (defined as being awarded a certificate of completion of training and entering the United Kingdom Specialist Register) prior to or during their PhD work were excluded. Potential participants were invited by e-mail on four occasions to take part in an interview. Upon agreeing to a time for the interview, participants were provided with an information sheet and asked for informed consent.

### Interviews

Interviews took place at a location that suited the participant, most frequently in an office or a quiet cafe; they lasted approximately 45 minutes and were audio-recorded and transcribed verbatim. The interviewer (V.F.R.) kept field notes during the interviews.

All interviews followed the structure set out in the interview guide, based on the findings of our scoping review as well as on the systems theory framework.^5,8^ Participants were asked open-ended questions about their careers to date, their experiences pursuing a PhD, and their career plans post PhD.

### Data analysis

**Figure 2:**
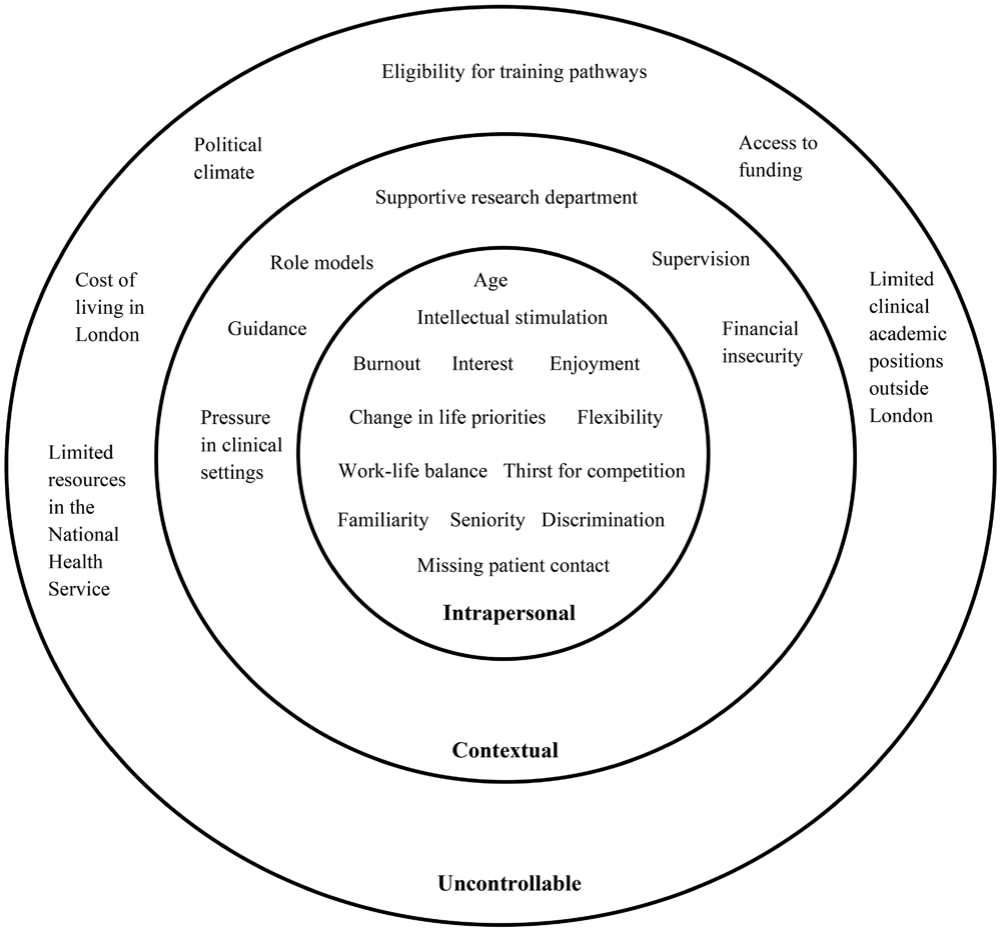
The intrapersonal, contextual, and uncontrollable influences on the postdoctoral career decision making of clinical academic physicians, University College London, 2015–2016, according to the systems theory framework.

We deidentified the interview transcripts and coded them using NVivo 11 (QSR International, Melbourne, Australia). Transcripts were initially divided into two groups, according to whether the participant was currently undergoing or had recently submitted her or his PhD thesis. As the two groups did not reveal starkly different narratives, we rejoined them and instead divided them into typologies according to participants’ reasons for pursuing a PhD. We then divided the factors that influenced these reasons according to whether they occurred within the individual (intrapersonal); within the individual’s social system (contextual); or within the individual’s broader environment or society (uncontrollable), per the systems theory framework (see Figure 2 for a map of the factors).

Our analytic approach combined induction (data-driven generalization) and deduction (theory-driven exploration of hypotheses).^11^ This approach complemented the study aim (to describe the influences on clinical academic physicians’ postdoctoral career decision making) by allowing the concepts at the core of the theoretical framework to be integral to the process of analysis, while at the same time allowing themes to emerge directly from the data. The transcripts were initially analyzed by one of the researchers (V.F.R.). Two different researchers (N.J.F., H.B.) then coded a sample of the transcripts; disagreements were discussed by the group and consensus was reached. Eight transcripts were shared between the research team who discussed the emerging themes. We adopted Braun and Clarke’s^12^ model of thematic analysis with a six-phase approach to generate, review, and define themes within the interview transcripts. Noting distinctive similarities or differences in recurrent themes between individuals allowed us to generate typologies. Finally, we checked the study design and reporting for compliance with the Consolidated Criteria for Reporting Qualitative Research checklist.^13^

## Results

In contrast to UCL’s overall gender distribution for physicians enrolled in a PhD program, our sample included more men (n = 21; 60%) than women (n = 14; 40%). Participants included both current doctoral trainees (n = 23; 66%) and trainees who had submitted their doctoral thesis during the previous year (n = 12; 34%). The stage of clinical training for participants from both of these groups ranged from the beginning of core medical training to the seventh year of subspecialty training (see Table 1). Data saturation may not have been reached in this latter group because of a low response rate in this population. However, when examining participants’ narratives by the differences and similarities in their experiences, we found no distinctions between these groups.

**Table 1:**
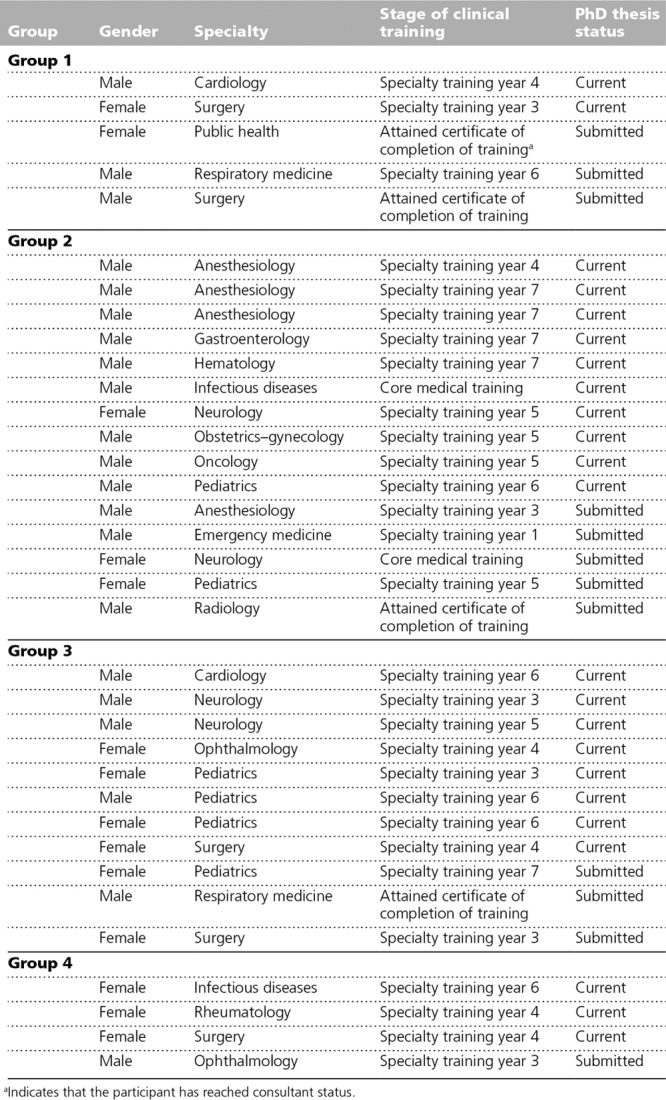
Demographic and Training Characteristics of 35 Clinical Academic Physicians Interviewed for a Study of the Influences on their Postdoctoral Career Decision Making, University College London, 2015–2016

From our data analysis, four prominent reasons for undertaking a PhD emerged: (1) to pursue a clinical academic career; (2) to complete an extensive period of research to understand whether a clinical academic career was the desired path forward; (3) to improve clinical career prospects; and (4) to take a break from clinical training. Stemming from these reasons, participants described their motivations for pursuing or not pursuing a position that combined both clinical and academic duties immediately after completing a PhD. We present these reasons and illustrative quotations by intrapersonal, contextual, and uncontrollable influences, as outlined in the systems theory framework (see Tables 2 and 3 for additional quotations and Figure 2 for a map of the influences).^8^

**Table 2:**
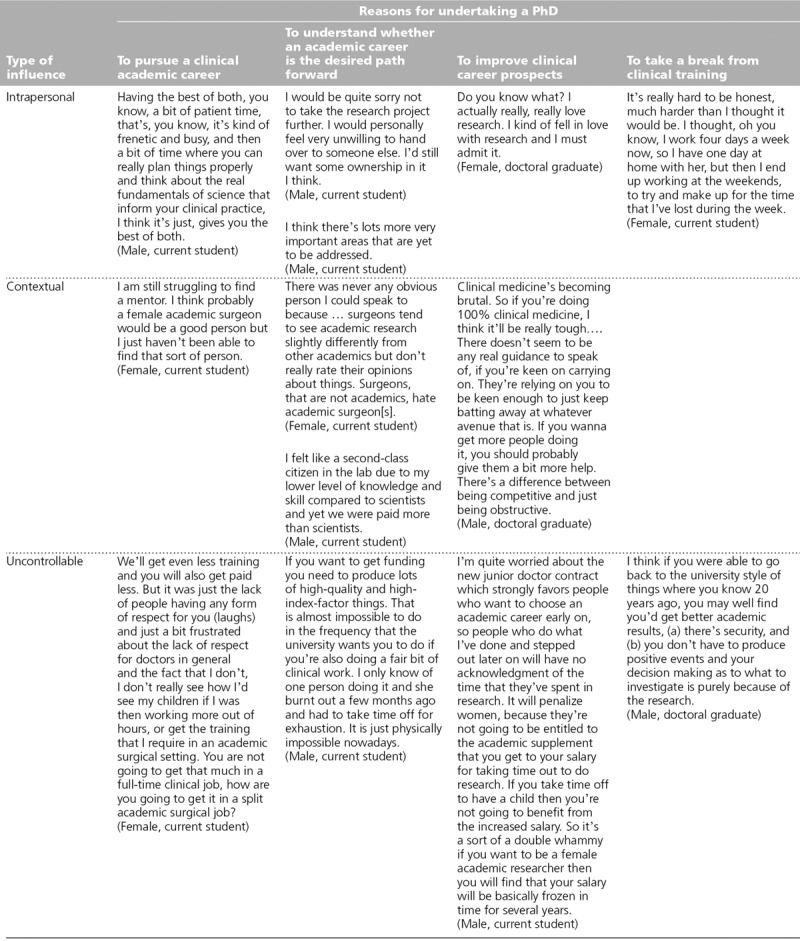
Quotations Describing the Intrapersonal, Contextual, and Uncontrollable Influences on the Postdoctoral Career Decision Making of 35 Clinical Academic Physicians, University College London, 2015–2016

**Table 3:**
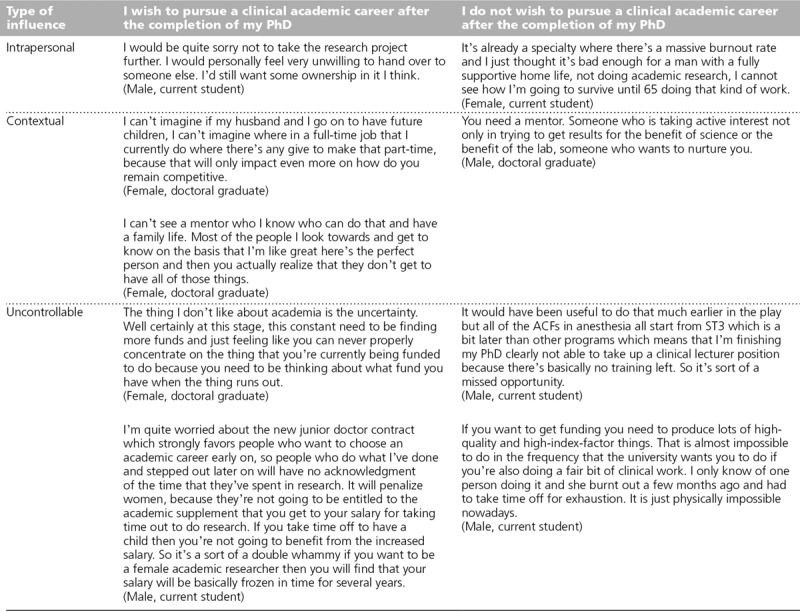
Quotations Describing the Intrapersonal, Contextual, and Uncontrollable Influences on the Postdoctoral Career Decision Making of Physicians Who Wish to Pursue a Clinical Academic Career Compared With Those Who Do Not, University College London, 2015–2016

### Undertaking a PhD to pursue a clinical academic career

Our first group included the five participants who exclusively chose to pursue a PhD to embark on a clinical academic career. All of these individuals planned to continue in research, having completed their PhD, because of intrapersonal influences, such as continued enjoyment and interest in their area of research. These participants found academia rewarding because of its ability to provide what clinical medicine no longer could, such as intellectual stimulation, competition, and diversity in their work. A career in clinical academia had the potential to shape these participants into better clinicians, as their ability to understand and conduct high-quality research, which they honed during their PhD training, could inform their clinical practice. Perceived difficulties, common to all trainees, were superseded by personal motivations that influenced this group’s decision to stay in academic medicine.

When I was in medical school everything was really amazing and you had many aspirations and the world was your oyster … and then you go into medicine and you realize what the job actually entails and that is the whole ward monkey bit. I think the academic side of things then gives you back the aspiration which is nice … going down the academic route I think for me gives me back the intellectual input that I wanted in the first place. I think without the academic side of things, I would feel a bit stale. (Female, current student)

### Undertaking a PhD to understand whether an academic career is the desired path forward

The primarily intrapersonal motivation for pursuing a PhD for those in our second group was to experience academic life and to use such experiences to decide whether they wished to pursue a clinical academic career. The desire to pursue a clinical academic position was not uniform in this group of 15 participants. Similar to the first group, 7 of these participants found the intrapersonal values they cherished, which were absent in clinical medicine, in academia. Identifying an area for improvement in research or clinical practice spurred these participants to continue in academia. Conversely, those who decided not to pursue a clinical academic career after their PhD expressed either a loss of interest in academia or a change in life priorities. A desire to complete clinical training was often fueled by a contextual need to prioritize either time spent with children or the wish to start a family. Embarking on an academic career was viewed as financially impractical to those starting a family, particularly in London.

Life in London isn’t that easy with a young family and particularly not starting out down the academic route where you basically take a bit of a pay hit because of the nature of academic jobs. (Male, current student)

### Undertaking a PhD to improve clinical career prospects

Our third group of 11 participants revealed that their motivation for pursuing a PhD was a contextual need to bolster their curricula vitae in preparation for interviews for a clinical consultant position or entry into subspecialty training. For most of these participants, their decision to stay in clinical academia was motivated by a newfound interest in research, and they viewed a full-time clinical career as an unviable option. In contrast, the desire of 3 participants to discontinue a clinical academic career was based on an intrapersonal need to not be as actively involved in research. These individuals expressed feeling burned out because of seemingly ceaseless, competing clinical and academic commitments. Feeling clinically “deskilled” (as they lost skills they once possessed with time spent out of the clinic), fed up, and too “old” to be a clinical trainee motivated other participants to return to the clinic full-time. This decision was also fueled by a contextual desire for job and financial security that was not dependent on outputs such as publications, as well as an intrapersonal perception that postdoctoral funding would be more easily attainable once one was fully clinically qualified.

What I’ve had over the last five, six years is every night there’s something different I could be doing on my laptop. I could be writing this. I could be writing that … there’s always the pressure and it’s not right. I haven’t got anymore give at the moment. At the moment, I’m just burnt out. I don’t want to do any more. (Female, doctoral graduate)

### Undertaking a PhD to take a break from clinical training

The primary motivator for pursuing a PhD for the four participants in our fourth group was to take a break from clinical medicine. To some, this break represented a much-needed pause from the exhausting obligations of clinical medicine. For others, however, it was an opportunity to experience something different from their usual clinical routine. Two participants, who decided to remain in clinical academia, expressed a desire to learn and understand more about their field. Furthermore, their distress over limited resources and an inability to provide thorough patient care within the National Health Service inhibited them from returning to full-time clinical work. Those who planned to return to clinical work recounted difficulty adapting from having senior status in the hospital to being an inexperienced researcher. Feeling unable to switch off and balance work with family commitments overwhelmed one participant’s coping mechanisms. While academia provided this participant with the flexibility to schedule her work around her family obligations, she expressed a lack of interest in pursuing grants and publications. Missing patient contact and having a strong desire to return to a familiar setting informed her decision to leave clinical academia.

It was like my solace that I’d go back to do, I’d do a clinic a fortnight, and I’d really look forward to doing it, because it’s somewhere where I felt comfortable and I felt at home, away from this weird science world that I didn’t understand. (Female, current student)

### Other influences for pursuing a clinical academic career

In addition to the reasons described above, we identified a set of universal contextual influences across groups. For instance, participants noted difficulty accessing information and guidance on training pathways and uncertainty about how to progress in a clinical academic career, particularly if they were ineligible for the integrated academic training pathway.

Furthermore, obtaining funding was a significant uncontrollable concern for all participants in this study and in our quantitative assessment.^7^ Securing funding was viewed as an increasingly competitive process, and participants spoke of a need for funding to bridge the gap between the end of their PhD training and the next stage of their career. This concern applied predominantly to those who both were ineligible to proceed to the next stage of postdoctoral funding, known in the United Kingdom as the academic clinical lectureship which offers substantial protected research time, and were not yet qualified for a more senior fellowship, known as an intermediate fellowship. Being unable to secure this funding or to receive genuine investment from a mentor, supervisor, or role model who could highlight future opportunities was viewed by participants as another substantial barrier to a clinical academic career.

Supervisors and peers were revered when they were supportive and actively interested in participants’ personal lives and career plans. Research departments were also viewed positively when they allowed for flexibility around time management to help support participants with child-rearing obligations. In contrast, female surgical trainees’ accounts revealed a different picture. They described gender-based discrimination centered on abusive comments regarding maternity and supervisors’ preferential treatment of male colleagues. Lastly, many participants (not just female surgical trainees) voiced their fears that the new junior doctor contract in the United Kingdom may financially penalize both female clinicians and those interested in pursuing an academic career, due to potential increases in working hours accompanied by a reduction in out-of-hours pay.^14^

## Discussion

### Key findings

Trainees described four key reasons for pursuing a PhD: (1) to pursue a clinical academic career; (2) to complete an extensive period of research to understand whether a clinical academic career was the desired path forward; (3) to improve clinical career prospects; and (4) to take a break from clinical training. Those who aspired to a clinical academic career prior to pursuing a PhD continued to do so as they neared completion of their degree. Sensing that the academic workplace shared their values, receiving support from a family member who was also a physician, and maintaining an interest in research all acted as buffers to the difficulties trainees perceived in locating funding and a role model.

In contrast, more than half of those trainees who entered a PhD program to understand whether they wanted to pursue a clinical academic career subsequently chose not to do so. These trainees expressed a loss of interest in research; a shift in life priorities such as child rearing and financial stability; and an anticipated absence of support in the future, whether monetary or supervisory. The majority of those who initially embarked on their PhD to improve their clinical career prospects chose to pursue a postdoctoral clinical academic career. This decision was based on a newfound interest in research and on a declining interest in a solely clinical career, given increasingly strict regulations and the new proposed junior doctor contracts that call for an increase in “standard” working hours and for basic, rather than out-of-hours, pay.

For a small group of trainees, a PhD represented a pause from the obligations of clinical medicine and an opportunity to experience something different from their usual clinical routine. Trainees who decided to remain in clinical academia overcame significant difficulties during their PhD that strengthened their desire to learn and understand more about their field. Nevertheless, the only two trainees in this group who planned to leave academia continued to have overwhelming parental obligations and were motivated by a strong desire to return to a more controllable setting where they felt they belonged.

### Comparison to previous research

Our study is the first to analyze the intrapersonal, contextual, and uncontrollable factors that influenced the career decision making of early-career clinical academic physicians in the United Kingdom around the time of their PhD completion as well as how those factors might be interrelated. In the absence of other published studies from the United Kingdom examining career decision making using a comparable cohort, we noted similarities in the types of influences described previously by our North American counterparts.^5^

However, prior to defining these similarities, there are substantial differences in clinical training between the medical training system in the United Kingdom and that in North America that have implications for this discussion. In the United States, for example, residency refers to the period of medical training completed after a graduate-level medical degree; it can last three to five years depending on specialty choice. In the United Kingdom, however, following completion of an undergraduate-level medical degree, physicians undergo a more extensive period of postgraduate clinical training. This training begins with a two-year foundation program followed by three to eight years of specialist training. It is in this latter stage that physicians are typically encouraged to enter academic training. In the United Kingdom, embarking on a PhD is the most common entry point into a clinical academic career, with many trainees also using their PhD as a way to distinguish themselves from their colleagues in their clinical career advancement.

The issues around the availability of funding and mentorship we identified were also identified in previous North American studies. Difficulties balancing work and family obligations and gender discrimination also appeared in the North American literature. However, missing from previous accounts but included in ours were an understanding of the different influences that may affect career decision making and how these may be interrelated as well as a recognition of the intrapersonal and political influences that play a central part in such career decisions. Additionally, in comparison with the findings from our earlier quantitative study, a greater proportion of trainees expressed a desire to pursue a clinical academic career, and nearly all trainees were familiar with a clinical lectureship, though fewer aspired to one.

### Strengths and limitations

Our study is the first to describe these influences using a theoretical framework. The systems theory framework provided us with a structure to understand these factors and how they may be interrelated. Additionally, classifying distinct groups of participants, according to their reasons for pursuing a PhD, enabled us to recommend interventions (see below). Another strength is our data analysis. The transcripts were initially analyzed by one of the researchers. A sample of these were then coded separately by two different researchers and, in the event of disagreements, discussed as a group.

Although our study included a wide representation of both medical and surgical specialties, our sample was composed of mostly male trainees, from one university, who self-selected to participate. Therefore, it is possible that participants’ responses may have reflected a biased characteristic prevalent in these trainees or exaggerated the strength of a particular influence. Nevertheless, trainees described both positive and negative experiences pursuing a PhD, with many desiring a clinical academic career afterward. Finally, participants did not review the themes prior to our submission of this article; however, they will be invited to provide feedback on the findings.

### Recommendations and directions for future research

Our study highlighted a need to target efforts at retaining clinical academic physicians according to their reasons for pursuing a PhD and their subsequent experiences with this process. For instance, those who embarked on a PhD primarily to pursue their interest in a clinical academic career continued to do so despite experiencing negative influences during their studies. More revealing, however, were the accounts of those who were interested in research but subsequently decided to leave academia. Implementing supervision quality checks by mandating graduate tutors or establishing contact with trainees on a frequent basis and providing timely and clear information on the support services available within the university may allow us to better track the environmental influences that trainees experience and the guidance they seek. Asking supervisors and heads of departments to openly encourage collaboration, supporting parents by enforcing flexible working hours, and guarding against discrimination by promoting managerial and diversity training are all essential to promoting an inclusive and supportive workplace. Furthermore, encouraging trainees to initiate mentoring relationships may help them feel more empowered.

These suggestions are not necessarily limited to the United Kingdom; many of the influences addressed by these suggestions also have been identified in studies in North America. Therefore, our recommendations are potentially generalizable to institutions located there. However, further quantitative and qualitative research into different educational contexts is required to confirm this generalizability. We cannot conclude whether any one motivation for pursuing a PhD, as cited by the trainees in our study, will lead to clinical academic success. However, a future study examining the eventual career progression of this cohort may reveal whether these factors continue to play a role in physicians’ career decision making.

Although our findings highlight the importance of intrapersonal influences, such as motivation, in career decision making, we found that such motivation could vary between the beginning and end of trainees’ PhD work. In some circumstances, such motivation was undermined by the influences mentioned above, while in others the motivation to pursue a clinical academic career only emerged as a consequence of PhD training. Across specialties, trainees often perceived, or were advised by senior physicians, that a PhD was required to improve their prospects of becoming a clinical consultant. Though trainees noted that such advice increased competition for already-limited funding, we recognize that it also motivated trainees who may not have previously considered academia to pursue a clinical academic career. Aware of funding limitations, we acknowledge a need for those responsible for overseeing clinical training to be well informed of the long-term benefits of training academically qualified physicians. We suggest providing support to those who are interested in a clinical academic career but are ineligible to continue in the prescribed National Institute for Health Research integrated academic training pathway. Finally, in light of current political uncertainty, universities, hospitals, and external agencies alike must increase their efforts to inspire and assuage early-career clinical academic physicians’ fears regarding their future in academia.

## Acknowledgments:

The authors would like to thank the trainees who took part in this study and the University College London administrative services staff who provided the list of doctoral trainees.
